# Generalized Pareto for Pattern-Oriented Random Walk Modelling of Organisms’ Movements

**DOI:** 10.1371/journal.pone.0132231

**Published:** 2015-07-14

**Authors:** Sophie Bertrand, Rocío Joo, Ronan Fablet

**Affiliations:** 1 Institut de Recherche pour le Développement (IRD), UMR212 EME, Centre de Recherche Halieutique Méditerranéenne et Tropicale, Avenue Jean Monnet, BP 171, 34203 Sète Cedex, France; 2 IMARPE, Esquina Gamarra y General Valle S/N Chucuito, Callao, Lima, Peru; 3 Institut TELECOM; TELECOM Bretagne; UMR CNRS 3192 Lab-STICC, Technopôle Brest Iroise CS 83818, 29238 Brest cedex, France; Ecologie, Systématique & Evolution, FRANCE

## Abstract

How organisms move and disperse is crucial to understand how population dynamics relates to the spatial heterogeneity of the environment. Random walk (RW) models are typical tools to describe movement patterns. Whether Lévy or alternative RW better describes forager movements is keenly debated. We get around this issue using the Generalized Pareto Distribution (GPD). GPD includes as specific cases Normal, exponential and power law distributions, which underlie Brownian, Poisson-like and Lévy walks respectively. Whereas previous studies typically confronted a limited set of candidate models, GPD lets the most likely RW model emerge from the data. We illustrate the wide applicability of the method using GPS-tracked seabird foraging movements and fishing vessel movements tracked by Vessel Monitoring System (VMS), both collected in the Peruvian pelagic ecosystem. The two parameters from the fitted GPD, a scale and a shape parameter, provide a synoptic characterization of the observed movement in terms of characteristic scale and diffusive property. They reveal and quantify the variability, among species and individuals, of the spatial strategies selected by predators foraging on a common prey field. The GPD parameters constitute relevant metrics for (1) providing a synthetic and pattern–oriented description of movement, (2) using top predators as ecosystem indicators and (3) studying the variability of spatial behaviour among species or among individuals with different personalities.

## Introduction

The characterization of foraging movements by random walk (RW) models (e.g. [1]) has received increased attention since GPS tracking made high-resolution records of animal and human displacements easily available (e.g. [2–4]). In such a probabilistic framework, movement is seen as a succession of elementary behavioural events called ‘moves’ [1] whose lengths are defined by random draws from a probability density function. While the dominant RW model in statistical physics has been the Brownian walk (characterized by a Gaussian behaviour of the tail of the move lengths distribution, and producing a normally diffusive behaviour), early studies in ecology suggested that a variety of foragers, from microzooplankton to humans (e.g. [5–10]), perform movements well described by Lévy walks. Lévy walks are characterized by the occurrence of rare and very large moves in the trajectory, producing heavy-tailed (power-law) move-length distributions, and revealing super diffusive spatial behaviour (e.g. [11, 12]). Regarding foraging behaviours, this super diffusive behaviour may emerge from the patchiness of the prey fields [13]. There has been a great deal of controversy regarding whether random walks should be phenomenological or mechanistic, and whether methods typically used to test the Lévy walk hypothesis are appropriate. [14–25].

One of the controversies originated in the modelling paradigm [2]: Should RWs provide a phenomenological, synthetic and pattern-oriented description of movement? This would justify the search for one global and parsimonious RW model for each study case. Or rather, should RWs provide a biomechanical, process-oriented description of movement? This would justify less parsimonious composite models possibly involving different RWs according to the activity in which the organisms are engaged at each move (e.g. state-space model approaches, [26] for seabirds, [27] for fishing vessels). Indeed, the two paradigms allow addressing different critical issues: while the process-oriented paradigm focuses on understanding the determinants of movement (e.g. bioenergetics or environmental constraints), the pattern–oriented paradigm may provide synthetic metrics of movement, highly valuable for studying the interactions between predators and their prey field (e.g. [28]), for studying some components of animal personalities (e.g. [29–31]) or for using top predator spatial behaviours as ecosystem indicators (e.g. [13, 32]).

A second controversy is linked to the methods that have been used to test the Lévy walk hypothesis from the power-tailed behaviour of the move length distribution. After graphical methods were proven inaccurate, the maximum likelihood criterion was recommended to perform an unbiased estimation of the parameter of the power-law distribution, and the comparison to alternative models according to the Akaike Information Criterion (AIC [33]) was encouraged [17,34]. Revisiting early works with this methodology and accepting/rejecting the AIC's best candidate model with a G-test for goodness-of-fit (GOF) test [35], Edwards *et al*. [16,24] and Edwards [23] stated that most of the original conclusions of Lévy walks in animal and human foraging strategies had to be overturned. A few cases could be described by the exponential distribution, a specific case of light-tailed distributions producing non-super diffusive spatial behaviours. Such exponentially distributed move lengths would correspond to purely random search strategies, i.e. Poisson distributed times for changes in search directions. In most cases, none of the tested models fitted the data, suggesting that the proposed candidate models were not appropriate.

Here, we argue that testing exponential vs. power-tailed distributions seems fairly restrictive to determine if super-diffusive or normally diffusive behaviours are dominant among foraging strategies. We propose Generalized Pareto distributions to model the movement of animal and human foragers as a random walk. This two-parameter class of distributions comprise as specific cases the Gaussian, exponential and power-law distributions, within a wider continuum of normally and super-diffusive RWs that allows getting around the endless debate opposing Lévy to other types of random walk models. Moreover, we consider that the G-test is not appropriate for evaluating the goodness of fit of such models because it requires binning continuous data, which is highly sensitive to the rules chosen for data quantization. We propose the use of the Cramér-von Mises test, exploiting directly the cumulative distribution of the original data, thus not requiring any binning. Finally, we address the problem of the determination of the start of the tail of the distribution, which is a critical issue for evaluating whether or not a Lévy walk hypothesis holds. Indeed, from a theoretical point of view, the super-diffusive behaviour of a Lévy walk [11, 12] is fully characterized by the asymptotic power-tailed behaviour of the distribution of the forager move lengths. Then, the tail of the distribution alone, and not its first percentiles, defines this super-diffusive characteristic. While explicitly addressed in the field of extreme event statistics [36], the determination of the start of the tail is an issue that has been ignored in the evaluation of the Lévy walk hypothesis in movement ecology. Power-tailed behaviour has typically been tested for a tail starting at the minimum value of the dataset or using histogram-based criteria that are very sensitive to the binning. To address this issue, we provide a pragmatic definition of the tail of the move length distribution.

Applying this methodology to the movements of two species of GPS-tracked seabirds and fishermen, we (1) illustrate the wide applicability of the proposed method on a variety of tracking data and (2) reveal inter-individual and interspecific variability in the spatial strategies selected by predators foraging on a common prey field.

## Method and Case Study Data

We consider the Generalized Pareto Distribution (GPD) to model the movements of living organisms, as described by the tails of their move-length distributions. A key feature of GPDs is to embed as special cases the Gaussian, exponential and power distributions, which underlie Brownian, Poisson-like and Lévy walks. The GPD is a two-parameter family of distributions [37, 38] with the following cumulative distribution function [39]:

$$F_{\sigma ,k}\left(l\right)=\{\begin{array}{l} 1-{\left(1+\frac{kl}{\sigma }\right)}^{-1/k},\;k\neq 0\\ 1-exp\left(-\frac{l}{\sigma }\right),\;k=0 \end{array}$$ (Eq 1)

and the probability density function:

$$f_{k,\sigma }\left(l\right)=\{\begin{array}{l} \frac{1}{\sigma }{\left(1+\frac{kl}{\sigma }\right)}^{-1-1/k},\;k\neq 0\\ \frac{1}{\sigma }exp\left(-\frac{l}{\sigma }\right)\;,\;k=0 \end{array}$$ (Eq 2)

where the *σ* > 0 and −∞ < *k* < ∞ are the scale and shape parameters; and the domain of x is [θ, ∞ [when *k* > 0 or [θ, *σ>*] when *k* ≤ 0; θ is the start of the tail of the distribution. The scale parameter, *σ*, characterizes the spatial range or characteristic scale of the movement patterns. The shape parameter *k* describes the asymptotic behaviour of the distribution; in other words, the relative contribution of long moves vs. short moves, i.e. the thickness of the tail that determines the diffusive property of the movement.

**Fig 1:**
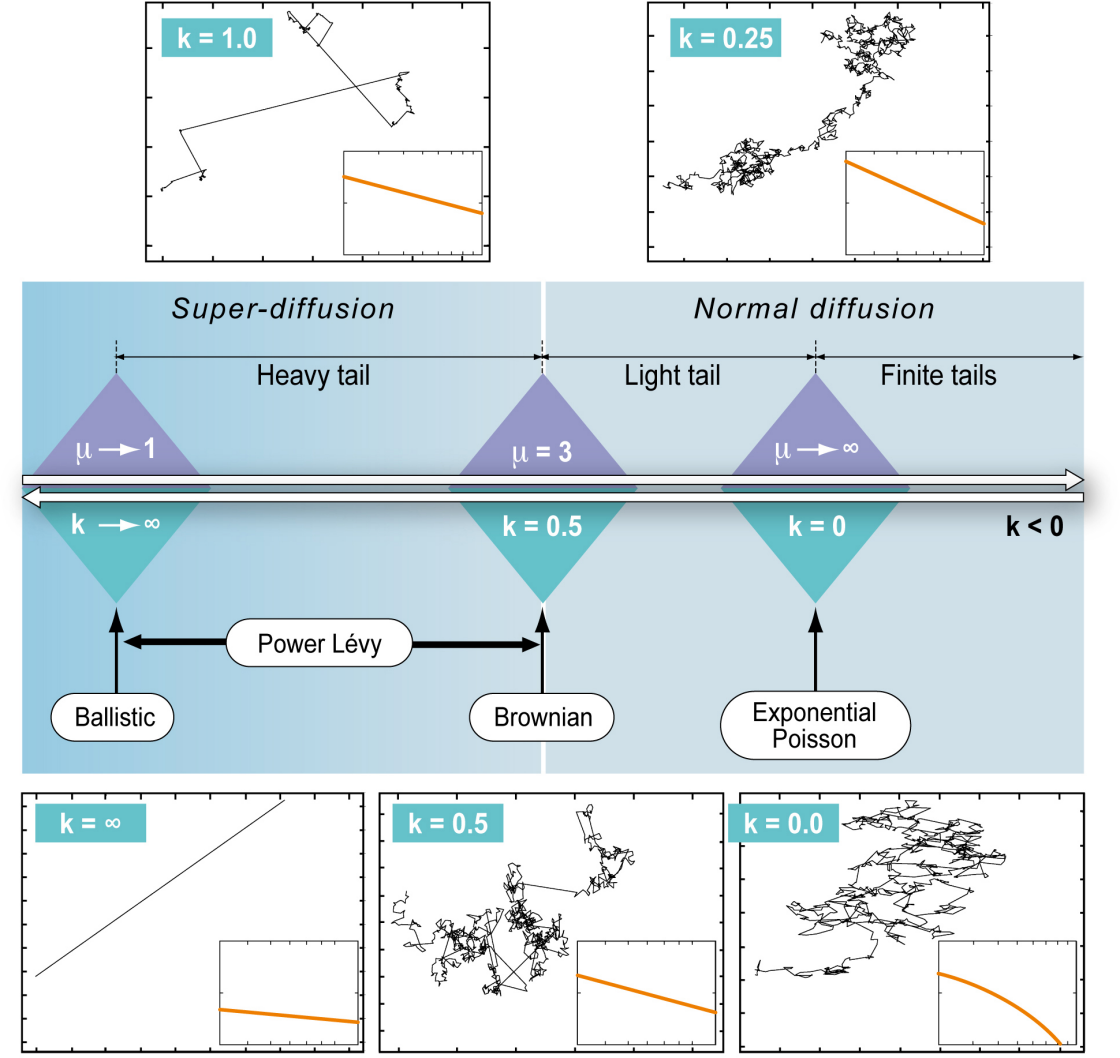
Generalized Pareto Distribution, a continuum from Exponential-Poisson to Power-Lévy walk patterns: parameter *k* of the GPD (Eqn.1) defines a continuum of distributions from light-tailed (*k*<0) to heavy-tailed (*k*>0.5). We show typical trajectories emerging from random realizations of those different move distributions, including a Poisson-exponential motion (*k* = 0), a Brownian-Gaussian motion (*k* = 0.5) and a Lévy-power walk (*k*>0.5) with *k* = 1. For each case, we also show in the lower right inset the log-log plot of the corresponding move length probability density function.

The thickness of the tail of a distribution is generally defined relative to that of a Normal distribution. A distribution that has a tendency to generate a higher proportion of extreme values than the Normal distribution is referred to as a heavy-tailed distribution. A distribution that has a tendency to generate a lower proportion of extreme values than the Normal distribution is referred to as a light-tailed distribution. The GPD comprises finite-tailed (*k*<0, e.g. Uniform and Beta distributions), light-tailed (*k* in [0; 0.5], e.g. exponential, gamma, or Gumbel distributions), heavy-tailed (*k* in] 0.5; ∞[, e.g. Lévy, Pareto, log-normal, Burr, Cauchy and log-gamma distributions) and ballistic (infinite *k* values) distributions. As illustrated in Fig 1, the exponential (*k* = 0) and Lévy walk (*k* > 0.5) hypotheses lie within this continuum of distributions. Parameter *k* is associated with the power-law (Lévy) exponent *μ* as *μ = 1+1/k*

Regarding the interpretation of forager movements in terms of diffusion, the reference point is the Brownian walk (*k* = 0.5), which distinguishes linear diffusive behaviour (*k*<0.5) from the super-diffusive behaviour exhibited by Lévy walks (*k*>0.5). The estimation of model parameters *k* and *σ* from move length observations is carried out using a maximum likelihood estimation. The log-likelihood of a sample of move lengths *l = (l*_*1*_,*l*_*2*_,*…l*_*n*_ *)* is given by [39]:

$$L\;\left(l,k,\sigma \right)=\prod_{i=1}^{n}f_{k,\sigma }\left(l_{i}\right)=-n\ln k+\ln\left(\Pi_{i=1}^{n}\left({\left(1+\frac{kl_{i}}{\sigma }\right)}^{-\frac{1}{k}-1}\right)\right)$$ (Eq 3)

The analytical maximization of the log-likelihood for GPD is not possible; hence numerical optimizations are carried out. In the present work, the maximization of the log-likelihood functions is performed with the Matlab toolbox « Statistics », that proceeds using a gradient descent initialized with the method of moments.

**Fig 2:**
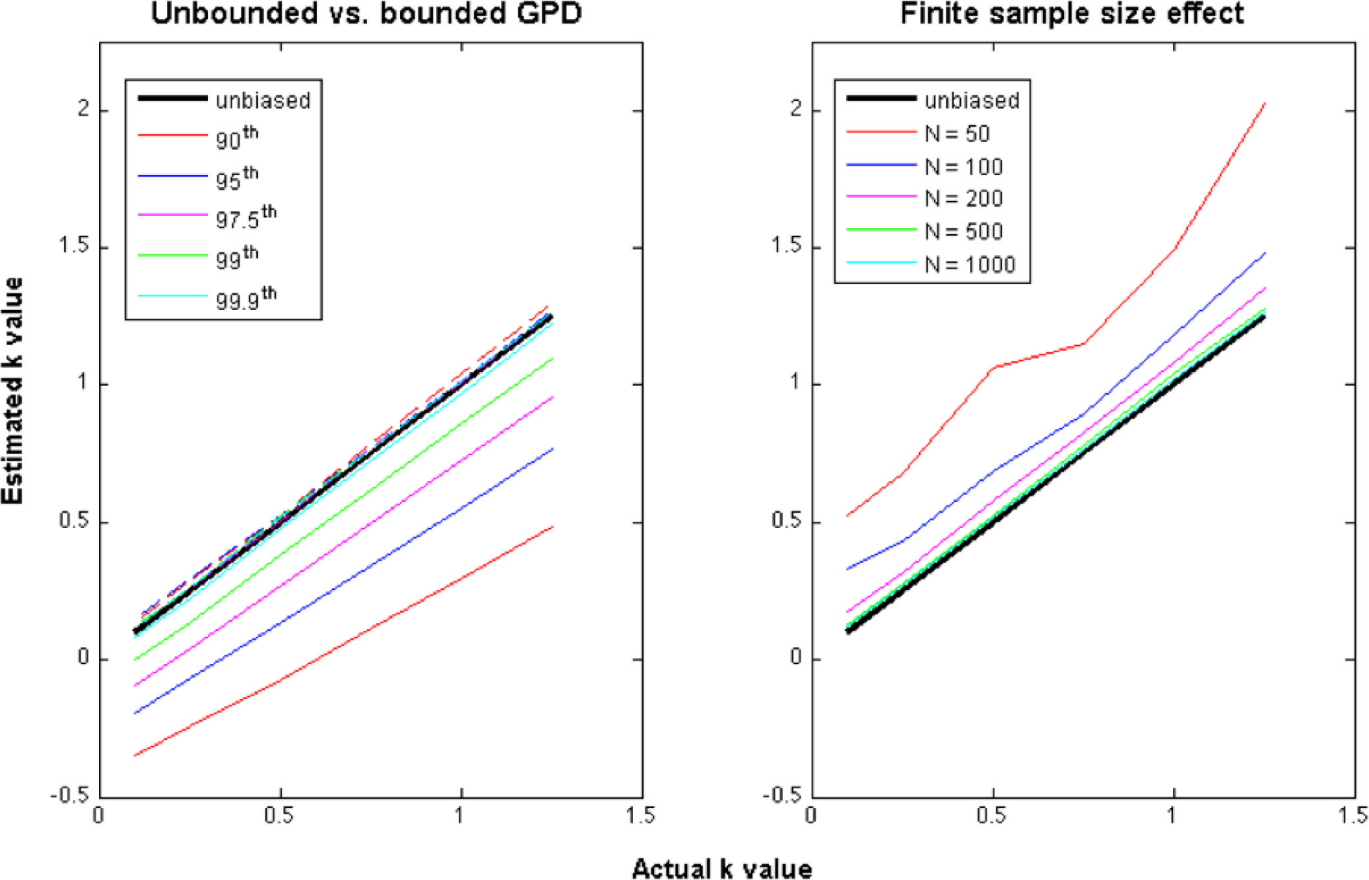
Analysis of the bias in the estimation of parameter *k* for both bounded and unbounded GPDs and for different sample sizes: mean estimation bias for the unbounded GPD (solid lines) and the bounded GPD (dashed lines) depending on the percentile of the observed maximal value (left), mean estimation bias for the bounded GPD as a function of the sample size N (right). In both cases, we report the zero-bias case (solid black line). The mean bias was estimated over 200 simulations.

From 200 simulations, carried out for values of *k* from 0.1 to 1.25, we analyse the estimation bias on model parameters that may emerge, as real foragers' datasets (1) cannot exhibit infinite move length values and (2) are samples of finite size. The effect of the finite move length values was explored with a sample of size 1000 and by setting maximum values of the move lengths between the 90^th^ and the 99.9^th^ percentile for the GPD (Fig 2, left panel). These simulations clearly point out a bias in the parameter estimation for the unbounded GPD when the maximal value of the sample is below the 99.9^th^ percentile. In contrast, the parameter estimation for the bounded GPD does not exhibit such a bias. As a consequence, the bounded version of the GPD (BGPD) should be preferred for examining real observation data. Then the effect of the samples of finite size was examined with a maximal value of the observed move length set below the 99.9^th^ percentile of the actual GPD. A finite sample size greater than 200 observations, although relatively small, leads to a significant reduction in the bias (Fig 2, right panel). Because the bias was linear with respect to the actual *k* value, a bias correction relationship was estimated. Simulations for other maximal values of the observed move length range led to similar conclusions. Linear bias corrections were then estimated as a function of the size of the sample set and of the percentile of the maximum move length value. These corrections were then applied when fitting bounded GPDs to seabirds and fishing vessel movement patterns.

The probability density function of the bounded version of the GPD (BGPD) is given by:

$$g_{k,\sigma ,l_{max}}=\frac{f_{k,\sigma }\left(l\right)}{F_{k,\sigma }\left(l_{max}\right)}$$ (Eq 4)

and the log-likelihood functions given a sample of move lengths *l = (l*_*1*_,*l*_*2*_,*…l*_*n*_ *)* is

$$L\;\left(g,k,\sigma \right)=\prod_{i=1}^{n}g_{k,\sigma }\left(l_{i}\right)={\left(\;F_{k,\sigma }\left(l_{n}\right)\right)}^{-n}\prod_{i=1}^{n}f_{k,\sigma }\left(l_{i}\right)={\left(\;F_{k,\sigma }\left(l_{n}\right)\right)}^{-n}\left[-n\ln k+\ln\left(\Pi_{i=1}^{n}\left({\left(1+\frac{kl_{i}}{\sigma }\right)}^{-\frac{1}{k}-1}\right)\right)\right]$$ (Eq 5)

Similar to the GPD, the maximization of the BGPD log-likelihood is performed numerically using a gradient descent scheme.

We use the Cramér-von Mises test [40], hereafter CVM, as a GOF-test to examine whether or not the observed data are consistent with the fitted model. The CVM statistic is defined as follows:

$$\omega_{n}^{2}=\frac{1}{12n}+\overset{n}{\underset{i=1}{\sum }}{\left(U_{i,n}-\frac{2i-1}{2n}\right)}^{2}$$ (Eq 6)

Where *n* is the size of the sample and *U*_*1*_ *= F(X*_*1*_ *)*, …, *U*_*n*_ *= F(X*_*n*_ *)*. *X* is the random variable representing the move length and *F(X*_*i*_ *)* is the value of the cumulative distribution function corresponding to the *i* ^*-th*^ rank statistic in the sample. The same statistic *ω* ^2^ can be calculated for a number of random draws of size *n* from the fitted model. The null hypothesis of the GOF test (observations are consistent with the fitted model) is rejected with significance level *α* whenever $\omega_{n}^{2}\geq \omega_{\alpha }^{2}$, where $\omega_{\alpha }^{2}$ is the upper *α*-quantile of the distribution of *ω* ^2^ over the random draws from the fitted model. This test does not require any prior binning of the continuous move length data and appears more appropriate than the G-test used in [23], especially when small samples are considered [41]. In addition, the CVM test relies on rank statistics that compare the cumulative density functions over their entire domain, and not only their maximum difference, as the Kolmogorov-Smirnov test does. This results in a greater robustness to test for the relevance of the candidate model given some observed samples, particularly when dealing with heavy-tailed distributions.

As mentioned above, the definition of the start of the tail is a critical issue, generally overlooked in previous studies. Here, we formally define the start of the tail as the minimum data value for which the GOF test is significant. By coupling model estimation and the GOF measure in this way, we guarantee a consistent and adaptive definition of the tail, ensuring the statistical consistency of the fitted model.

As case studies, we consider two datasets collected in the Peruvian coastal upwelling ecosystem on anchovy (*Engraulis ringens*) main predators: seabirds and the purse seine fishery. In such pelagic ecosystems, water masses and fish schools are constantly moving [42] so that precise prey localization is unpredictable regardless of the predator, human or animal [8]. Foraging movements for all predators aim at the same goal: dealing with uncertainty in prey localization and maximizing encounters. Yet, they may adopt distinct forms due to differences in the cognitive, locomotory and motivational states of each predator. Synthetic metrics like the scale and shape parameters from the BGPD may be very useful for quantifying and comparing alternative spatial strategies.

The first dataset consisted of the movements deployed during four foraging trips by two seabird species, boobies (*Sula variegata*) and guanay cormorants (*Phalacrocorax bougainvilli*) from Isla Pescadores, a colony located off Lima, Peru (11°47’12” South, 77°14’25” West), where the two species breed in sympatry (authorizations for the fieldwork were delivered by SERNANP, Peru; no animals were handled directly for this study as data were collected from previous works). Their positions were recorded each second with miniaturized GPS [see 43 for protocol details; raw data available in Supporting Information S1, S2, S3 and S4 Dataset]. The original positions were resampled into moves (see Supporting Information S1 Protocol), where moves are defined as elementary behavioural events [1]. We fitted a BGPD, and alternative classical models for comparison, to the distribution of the move lengths observed for each foraging trip, each of them gathering more than 500 moves.

The second dataset consisted of the movements deployed by three industrial anchovy purse seiners, as monitored by the Peruvian Vessel Monitoring System (VMS [13, 8]; between 4 and 18° South and 72 and 82° West). VMS provides approximately one position per hour for each vessel. The original positions of fishing trips are re-sampled into moves ([1, 13, 8]; see the electronic supplementary material]. Since fishing trips in this fishery last on average 24h and VMS sampling is about 1 per hour, each trip is described by ~ 24 positions, producing less than 20 moves. Since model fits based on samples with less than 200 moves should not be considered, we fit a BGPD (and alternative models for comparison) to the yearly distributions of the move lengths of each vessel, considering that a given fisherman should exhibit some consistency in its spatial behaviour over time. These vessel data were already analysed in previous RW works [8, 23] and were selected for potential comparisons of the results.

## Results

### 3.1 Seabirds

**Fig 3:**
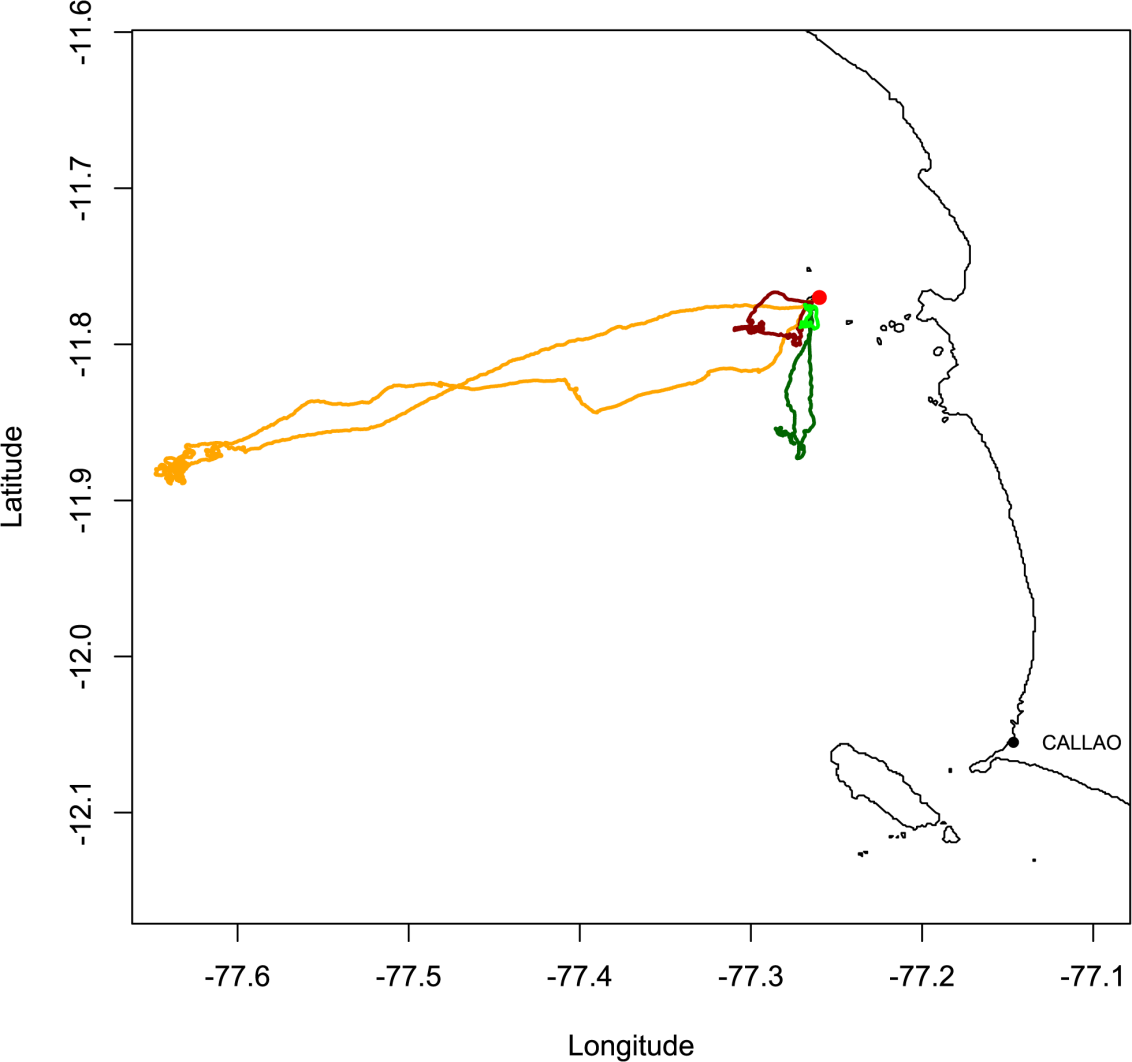
Foraging trips by four seabirds from a breeding colony in the Peruvian coastal waters (Pescadores Island, red dot) in November 2010. Trips were recorded with miniaturized GPS, providing one position every second. The orange and dark red tracks are from boobies (*Sula variegata*) and the dark and light green tracks are from cormorants (*Phalacrocorax bougainvillii*).

The four seabird foraging trips studied are presented in Fig 3. They lasted between ~ 1 and 4 hours with covered distances between 11 and 140 km, and maximum distances from the colony (ranges) between 2 and 43 km. Table 1 compares for each trip, through AIC, the fit of the exponential, power, Generalized Pareto and Bounded Generalized Pareto distributions to the move length data. The estimated shape parameters (expressed as $\hat{k}\;or\;\hat{\mu }=\frac{1}{1-\hat{k}}$) show that the four tracks are super diffusive and well described by Lévy walks ($\hat{k}\in ]0.5;\infty [$ or $\hat{\mu }\in ]1;3[$). With the proposed adaptive tail definition, BGPD was the model that allowed gathering the largest number of moves in the tail (50%-70% of the moves, other model results are not shown here).

**Table 1:** Model comparison on four seabird GPS-tracked foraging trips. For each trip, four alternative models are considered: Exponential (*f*(*l*)∼*l* ^−*μ*^), Power (*f*(*l*)∼*e* ^−*λl*^), Generalized Pareto (GPD), Bounded Generalized Pareto (BGPD), see Edwards (2011) for the likelihood functions of the first two models. Model parameter estimates $\hat{k},$ $\hat{\mu }=\frac{1}{1-\hat{k}}$, and $\hat{\sigma }$ are given except for the exponential model and the likelihood of the models are compared with the Akaike information criterion (AIC). The last column provides the total number of moves obtained after resampling the original track positions (N) and the % of moves retained in the tail, using the adaptive criteria defining the start of the tail as the minimum data value for which the GOF test of estimated model parameters is significant (CMV p-value >0.05).

Seabird [# moves; % moves in tail]	Model	$\hat{k}$ [$\hat{\mu }$]	$\hat{\sigma }$.10^−3^	AIC	CMV p-value
Peruvian booby (a) [1003; 50%]	Exponential	-	-	5723	<0.001
	Power	1.47 [1.68]	-	5720	<0.001
	GPD	0.53 [2.89]	58.52	5618	0.249
	**BGPD**	**0.67 [2.49]**	**55.32**	**5610**	**0.720**
Peruvian booby (b) [645; 70%]	Exponential	-	-	2970	<0.001
	Power	1.12 [1.89]	-	2690	0.001
	GPD	0.83 [2.21]	10.26	2680	0.102
	**BGPD**	**0.90 [2.11]**	**10.03**	**2680**	**0.105**
Guanay cormorant (c) [1858; 70%]	Exponential	-	-	7280	<0.001
	Power	1.61 [1.62]	-	4530	0.001
	GPD	1.69 [1.59]	0.78	4530	0.053
	**BGPD**	**1.85 [1.54]**	**0.76**	**4520**	**0.072**
Guanay cormorant (d) [1035; 70%]	Exponential	-	-	3420	<0.001
	Power	1.41 [1.71]	-	2230	0.143
	GPD	1.43 [1.70]	0.76	2230	0.127
	**BGPD**	**1.54 [1.65]**	**0.73**	**2220**	**0.180**

**Fig 4:**
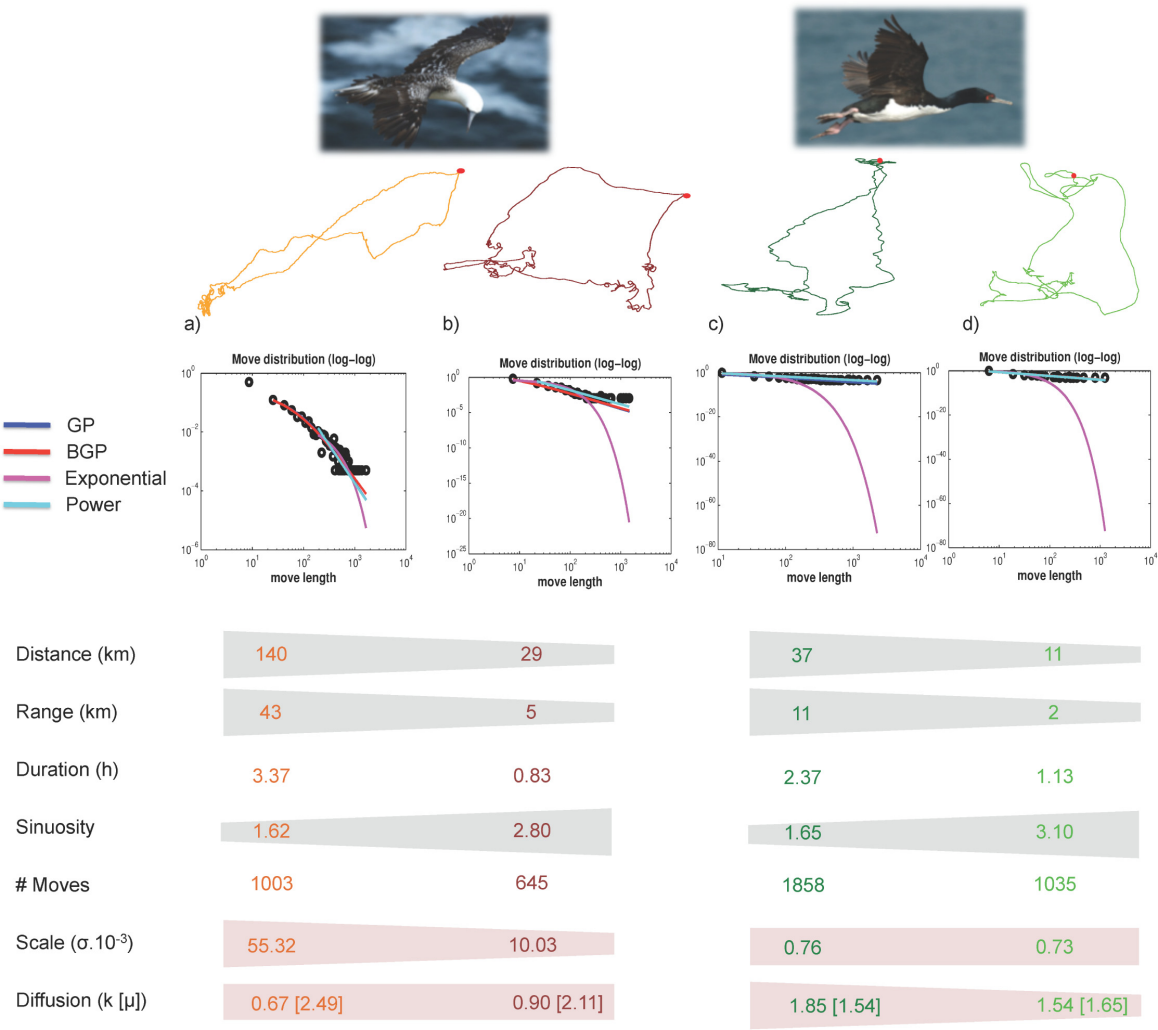
Synoptic characterization of the seabird foraging trips. Original tracks, log-log plots of the fits for the different candidate models and synthetic statistics characterizing the trips: Total distance covered during the foraging trip (km), maximum range from the colony (km), duration of the trip (h), global sinuosity of the trip (defined as distance/2*range), number of moves after resampling (cf. SI S3), scale parameter (*σ*) and shape parameter (*k*, describing diffusion) from the fitted BGPD.

The four track examples (Fig 4) illustrate that a diffusion coefficient alone does not allow comparing the area that two foragers may visit during a trip: diffusion coefficients are directly comparable only if the scale parameters are similar. For instance, booby *a* and cormorant *d* have comparable number of moves (*n* equal to 1003 and 1035, respectively) and the former has a lower diffusion coefficient than the latter (*k* equal to 0.67 and 1.54, respectively). However, booby *a* travelled farther distances than cormorant *d* (ranges of 43km and 2 km, respectively). Regarding individuals within the same species, boobies *a* and *b* show great differences in covered distance and range. Since the diffusion parameter k takes similar values for both boobies, it is the scale parameter *σ* (5.5.10^−2^ and 1.10^−2^ for boobies *a* and *b*, respectively) that conditions the characteristic length of the trajectory and the scope of exploration of the seabirds. Cormorants *c* and *d* show smaller covered distances and range values, with the former one taking larger values than the latter. In this case, scale parameters are comparable (7.6.10^−4^ and 7.3.10^−4^, respectively), and it is the difference in the diffusion parameter (1.85 and 1.54, respectively) that conditions the scope of exploration (ranges of 11 and 2 km, respectively).

### 3.2 Fishing vessels

**Fig 5:**
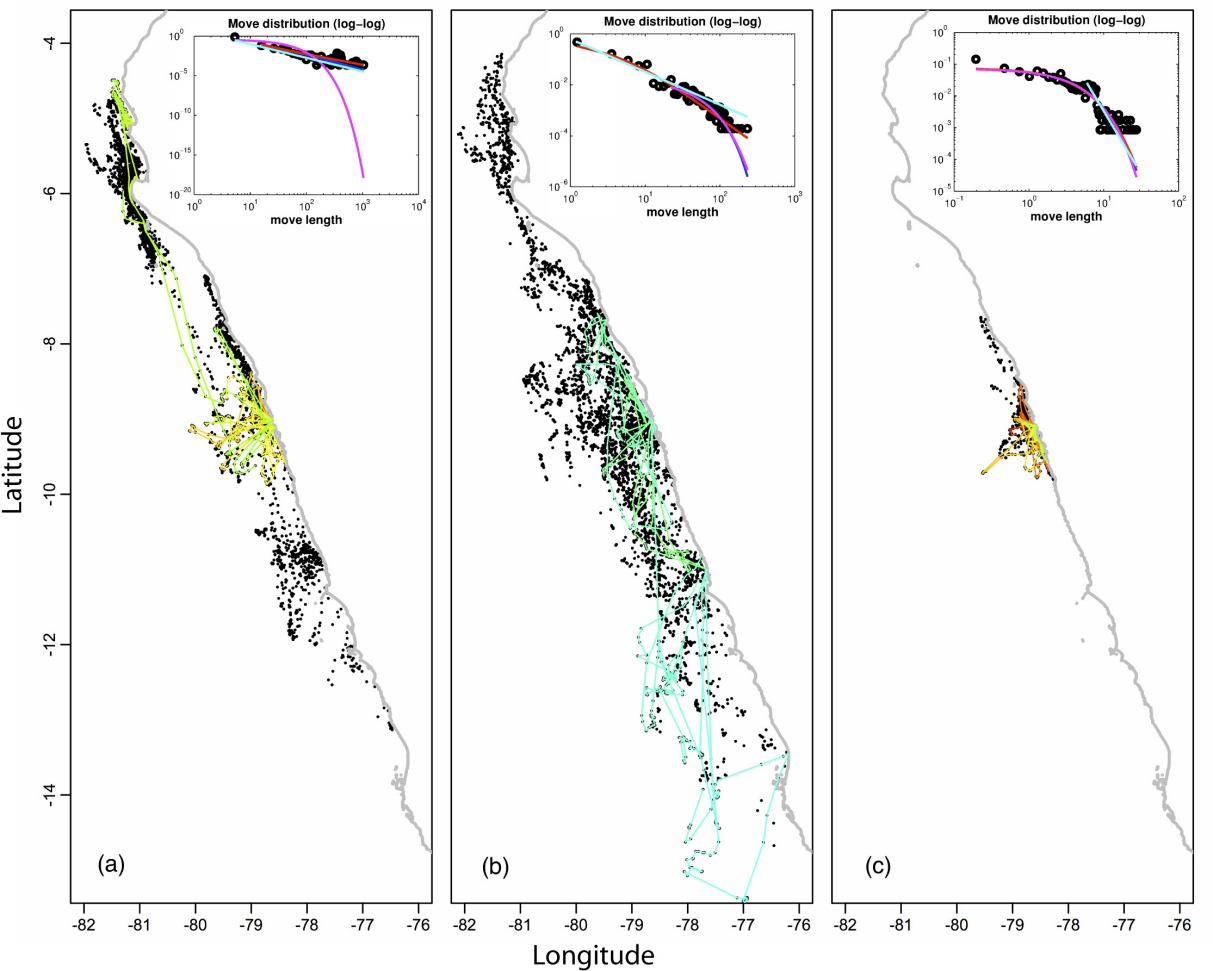
Fishing trips from anchovy purse seiners *a* (left panel), *b* (central panel) and *c* (right panel). Black dots represent the global set of positions for fishing trips deployed during one year. A sample of 25 fishing trips is represented with colored lines. The inset gives the log-log plot of the total move length distribution together with the fits of the four candidate models (Generalized Pareto in dark blue, Bounded Generalized Pareto in red, exponential in pink and power in light blue).

The fishing trips from the three anchovy purse seiners are presented in Fig 5. Bertrand et al. [8], based on graphical estimation methods, concluded that the movements of the three vessels were well described by Lévy walks, vessel *a* being the most diffusive ($\hat{\mu }=1.43$) and vessel *c* the least diffusive ($\hat{\mu }=2.43$). Edwards [23] on the same dataset concluded that although bounded Lévy was the most plausible model for vessels *a* and *b*, and exponential for vessel *c*, no model actually passed the G-GOF test. The conclusion was that these datasets did not conform to any candidate models. Table 2 compares for each vessel the fitting of the exponential, power, Generalized Pareto and Bounded Generalized Pareto distributions to the move length distributions. In the three cases, BGPD presented the minimum AIC, regardless of the rule chosen for defining the start of the tail of the move length distribution (i.e. either based on histograms as in previous works or using the proposed adaptive definition of the tail). Consistently with [8], vessels *a* ($\hat{k}=2$) and *b* ($\hat{k}=0.86$) are well described by a super diffusive Lévy walk behaviour ($\hat{k}\in ]0.5;\infty [$ or $\hat{\mu }\in ]1;3[$); in contrast, vessel *c* ($\hat{k}=0.05$) approaches a normally diffusive RW ($\hat{k}<0.05$), close to a Poisson-like (exponential tail) movement. The three vessels also differed in the characteristic length of the moves they deployed: vessel *a* exhibited the longest ($\hat{\sigma }$ = 25.29), and vessel *c* the shortest ($\hat{\sigma }$ = 3.31) moves. Since both the scale and shape parameters changed in the same direction, vessels *a* and *c* exhibited the widest and smallest scope of exploration respectively.

**Table 2:** Model comparison on VMS-tracked trips from three fishing vessels. For each vessel, four alternative models are considered: Exponential (*f*(*l*)∼*l* ^−*μ*^), Power (*f*(*l*)∼*e* ^−*λl*^), Generalized Pareto (GPD), Bounded Generalized Pareto (BGPD), see Edwards (2011) for the likelihood functions of the first two models. Model parameter estimates $\hat{k},$ $\hat{\mu }=\frac{1}{1-\hat{k}}$, and $\hat{\sigma }$ are given except for the exponential model and the likelihood of the models are compared with the Akaike information criterion (AIC). The last column provides the total number of moves obtained after the resampling of the original track positions (N) and the % of moves retained in the tail. Two protocols for the definition of the beginning of the distribution tail are compared: the empirical protocol used in previous works on the same data (Bertrand *et al*. 2005, 2007; Edwards 2011; the tail is defined based on the histogram of the move length built with Scott’s rule, excluding the first bin); the adaptive protocol proposed here defining the start of the tail as the minimum data value for which the GOF test of estimated model parameters is significant (CMV p-value >0.05). Note: AIC are not directly comparable when the tail definition is different because the observation sample is not the same.

Vessel [Number of moves ; % moves in the tail]	Model	$\hat{k}$ [$\hat{\mu }$]	$\hat{\sigma }$	AIC	CMV p-value
Vessel *a* Empirical tail [1224]	Exponential	0 [∞]	-	6 490	<0.01
	Power	1.39 [1.72]	-	5 830	<0.01
	GPD	1.54 [1.65]	9.17	5 830	<0.01
	**BGPD**	**3.45 [1.29]**	**7.56**	**5 750**	**0.026**
Vessel *b* Empirical tail [3498]	Exponential	0 [∞]	-	12 020	<0.001
	Power	1.67 [1.60]	-	11 770	<0.001
	GPD	0.67 [2.49]	4.84	11 360	0.026
	**BGPD**	**0.79 [2.26]**	**4.64**	**11 340**	**0.039**
Vessel *c* Empirical tail [824]	Exponential	0 [∞]	-	1 863	0.128
	Power	1.25 [1.80]	-	2 058	<0.001
	GPD	0.02 [42.14]	3.44	1 864	0.122
	**BGPD**	**0.04 [24.92]**	**3.41**	**1 863**	**0.113**
Vessel *a* Adaptive tail [872; 20%]	Exponential	0 [∞]	-	4 880	<0.001
	Power	1.54 [1.65]	-	4 880	<0.001
	GPD	1.02 [1.98]	27.77	4 656	0.013
	**BGPD**	**2 [1.50]**	**25.29**	**4 612**	**0.079**
Vessel *b* Adaptive tail [4204; 80%]	Exponential	0 [∞]	-	13 930	<0.001
	Power	2.17 [1.46]	-	13 770	<0.001
	GPD	0.73 [2.37]	3.89	12 990	0.053
	**BGPD**	**0.86 [2.16]**	**3.72**	**12 960**	**0.082**
Vessel *c* Adaptive tail [1054; 90%]	Exponential	0 [∞]	-	2 364	0.272
	Power	2.38 [1.42]	-	2 897	<0.001
	GPD	0.03 [28.96]	3.34	2 365	0.224
	**BGPD**	**0.05 [19.7]**	**3.31**	**2 364**	**0.220**

## Discussion

The visual inspection of tracks is usually the first step that an ecologist undertakes when studying foraging movements. Considering the seabird tracks studied here, this step immediately reveals that booby *a* went much farther from the colony than booby *b* and cormorants *d* and *c*. Basic statistics (range, distance covered) confirm and quantify this global pattern. Fitting BGPDs to these tracks provided additional and non-redundant information that was not accessible to visual inspection and basic statistics.

First, the estimated shape parameter ($\hat{k}$) revealed that all four tracks were well described by Lévy walks, suggesting that these four foraging movements were super diffusive. Anchovy is a pelagic gregarious forage fish, organized in hierarchical aggregations (schools, clusters of schools, cluster of clusters; see [44]), which are constantly moving (up to 26 km per day, [42]). Predators foraging on such prey fields constantly need to track this hierarchical organization, and this is certainly the reason why their foraging trajectories mix long moves (moves between anchovy aggregations) with short ones (moves within anchovy aggregations), producing super-diffusive patterns in their movements [13, 8].

Second, the fitted shape and scale parameters of the BGPD revealed that the studied boobies and cormorants resorted to different strategies while foraging the same prey field: boobies used moves with longer characteristic length (scale parameter) while cormorants used a more diffusive spatial strategy (larger shape parameter). The larger characteristic length of boobies’ moves may be linked to their morphology: *Sulids* are in general described to be optimal for fast and far flights [45]; *Sula variegata* in particular, when facing competition for food (including the fishery, [43]), possibly take advantage over cormorants from its greater foraging range [45]. The more diffusive patterns of cormorants emerge from a more contrasted contribution of short moves versus long moves, producing a skewed distribution of move lengths. Cormorants tend to rely more on collective foraging than boobies do. They form dense rafts when shoals are located, with large unbroken columns of flying individuals from the colony to the feeding ground [45, 46]. The importance of social information for cormorants may explain their more diffusive foraging movements: while involved in a column, cormorants fly straight to a fish aggregation producing long directed moves in contrast to much shorter moves while actively foraging on a fish aggregation. Such speculative interpretation obviously calls for complementary studies analyzing a much larger sample of tracks.

Here our main contribution is a BGPD-based methodology that (1) quantified differences among the studied tracks that were not trivial from visual inspection and basic statistics and (2) raised questions and hypotheses about differences in the foraging strategies adopted by two sympatric species foraging on the same prey field. The inference of BGPD parameters for a variety of scenarios of prey abundance and distribution should be explored to investigate how the two species deal with the great variability of the anchovy distribution (e.g. [47]), and may provide new insights on the behavioural niches of these two sympatric species [48].

The second case study, the movements of fishing vessels, provided a complementary illustration of the possibilities opened by the proposed methodology. Using the BGPD model, we characterized the different strategies exhibited by three fishermen. Similar to the seabirds, they all faced the need to track down the gregarious and constantly moving distribution of anchovy aggregations. Two of the vessels exhibited a super diffusive behaviour, well described by a Lévy walk, and the third one was best described by a normally diffusive behaviour. In that case study, the characteristic length of the observed moves was positively correlated to the diffusion coefficients, producing large differences in their scope of exploration.

Several hypotheses could explain such variability in the fishermen’s strategies, including a difference in their personalities. Allen & MacGlade [49] for instance showed that facing fluctuating fish conditions, several types of spatial behaviours may emerge among fishermen, namely, stochastic versus Cartesians, characterized respectively as hunters or risk takers, and followers or low risk takers. Such hypotheses should be evaluated in future works on larger samples of fishermen, allowing a thorough analysis of fishermen personalities in this fishery, and to evaluate the benefit, at the fishery scale, of mixing such personalities for discovering the location of new fish aggregations and optimally exploiting the prey field.

Overall, the proposed BGPD framework is applicable to any move length distribution, regardless of the forager trajectory under consideration. It provides a phenomenological, compact, and pattern-oriented description of movement. It quantifies informative and non-trivial components of foraging strategies: their characteristic size (*σ*, the scale parameter) and their diffusive behaviour (*k*, the shape parameter). It is parsimonious because it does not call for testing a battery of putative models, sometimes difficult to select among (e.g. [50]). In contrast, it lets the most likely random walk model emerge from the data. This model, along with the proposed adaptive tail definition, is a generic tool to analyse GPS-tracking data and foraging strategies. It allows quantitative and synoptic comparisons between foragers as well as detections of changes with time in the behaviour of a single forager. This methodological framework is a key step to test and validate hypotheses on the processes underlying the variability of foraging movements. As such, it is of critical importance in a variety of movement ecology issues, including for instance the use of the spatial behaviour of top predators as an ecosystem indicator (e.g. [32, 13, 51]), the comparison of the behavioural niche of different species foraging the same prey field (e.g. [48]) and the identification of different personalities within the same species (e.g. [29, 30, 31]).

## Supporting Information

S1 Dataset Raw data for booby a. (TXT)

S2 Dataset Raw data for booby b. (TXT)

S3 Dataset Raw data for cormorant c. (TXT)

S4 Dataset Raw data for cormorant d. (TXT)

S1 Protocol Resampling observed positions into moves. (DOCX)
