# Supplementary material for: Generalized Pareto for Pattern-Oriented Random Walk Modelling of Organisms’ Movements
Source: PLoS One. 2015 Jul 14;10(7):e0132231. doi: 10.1371/journal.pone.0132231 (PMC4501722; doi:10.1371/journal.pone.0132231)
Supplement: S1 Protocol — (DOCX) [file pone.0132231.s005.docx]

**Supporting Information**

**S5 Protocol**

**Resampling observed positions into moves**

Moving animals trace continuous paths through space. However, paths are usually digitalized discretely, by recording the spatial coordinates of the organism at regular time intervals and then connecting successive positions with straight lines. Such displacements performed during a regular time interval are called ‘steps’. Steps are an artificial discretization of the path that does no fit to any real behavioral event. If the sampling interval is too long, several behavioral events may be mixed inside a same step (undersampling); if the sampling interval is too short, a single behavioral event may be split into several steps (oversampling). There is no option for correcting undersampling, but oversampling can be corrected by resampling at lower resolution.

The simplest approach to resampling is to use the processing power of the human brain (Turchin, 1998), i.e. discrete time steps are aggregated by eye into moves. Such a procedure is subjective and may be affected by biases of the investigator, and is not adapted to the treatment of numerous paths.

In our two case study, the criteria we chose to resample original positions into moves was based on a threshold on turning angles between successive steps and empirically chosen based on (1) a good agreement with what could had been done by eye and (2) a single threshold value providing the best compromise between tracks of the same origin (VMS or GPS). We ended up with the following criteria: for vessels (VMS tracks, 1h period, see fig. 1) a new move was defined when a change in direction between two consecutive steps exceeded 10º; for seabirds (GPS tracks, 1s period, see Fig.2) a new move was defined when a change in direction between two consecutive steps exceeded 11.25º (=pi/16);

**
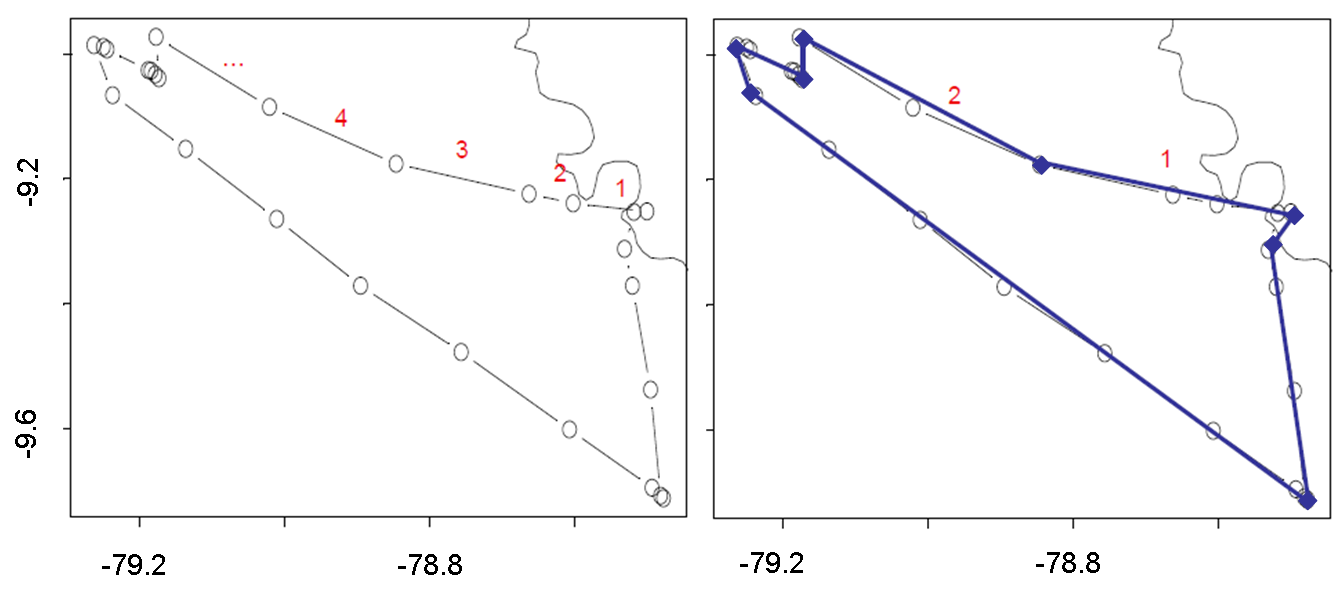
**

**Figure 1. Example of a vessel track observed at regular sampling intervals (left panel) and the same vessel track resampled into moves (right panel) with the empirical criteria described above.**


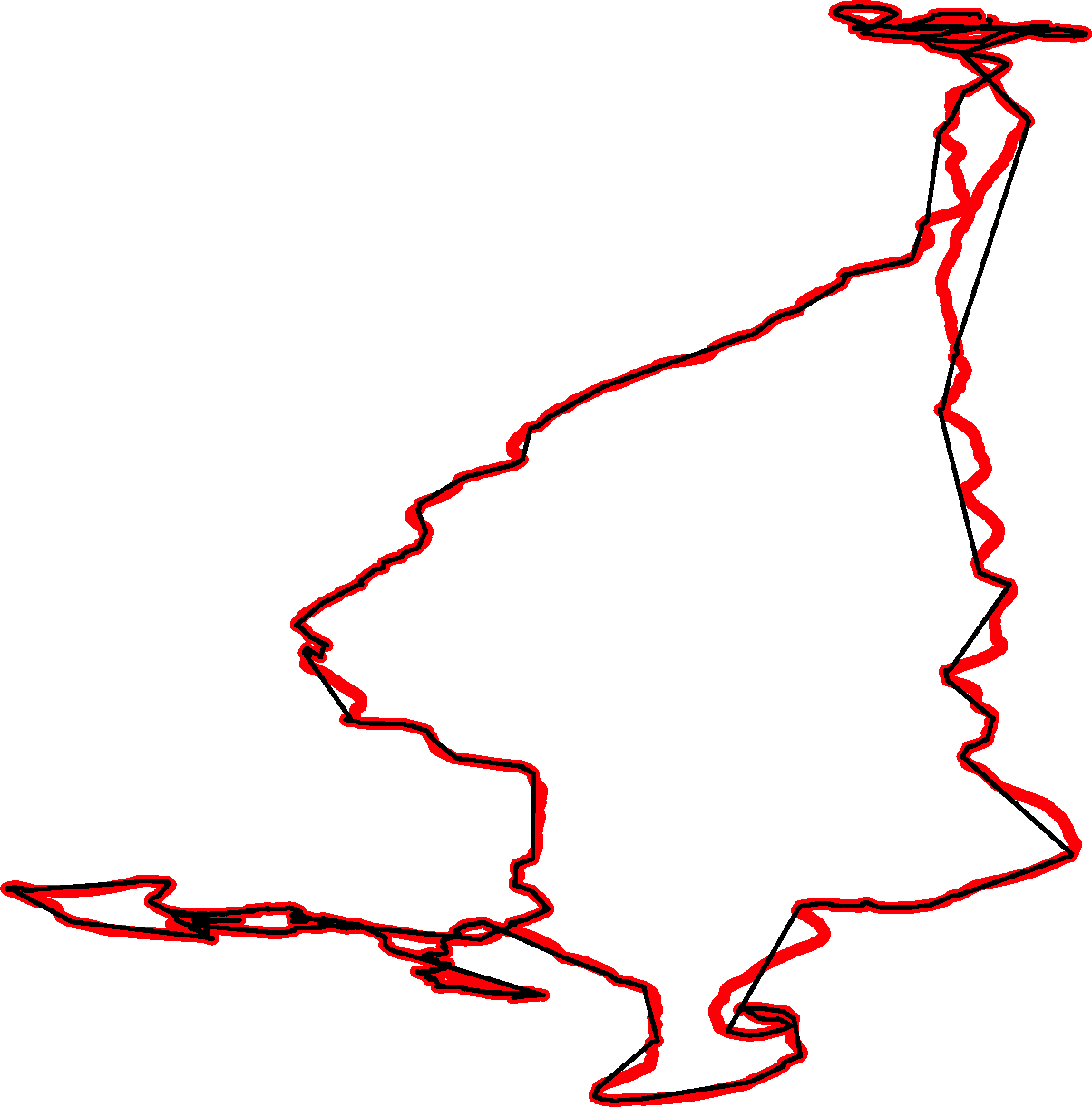

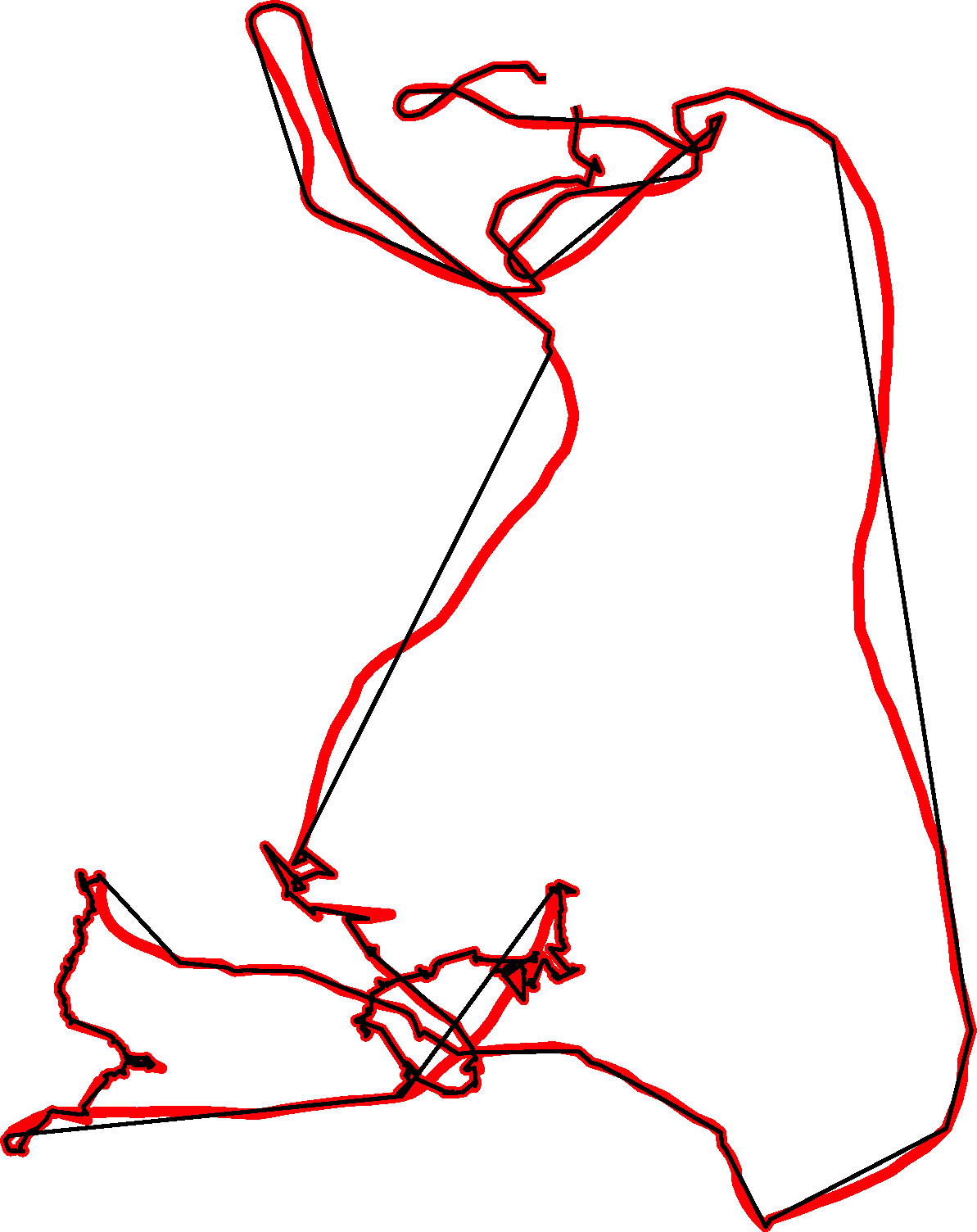

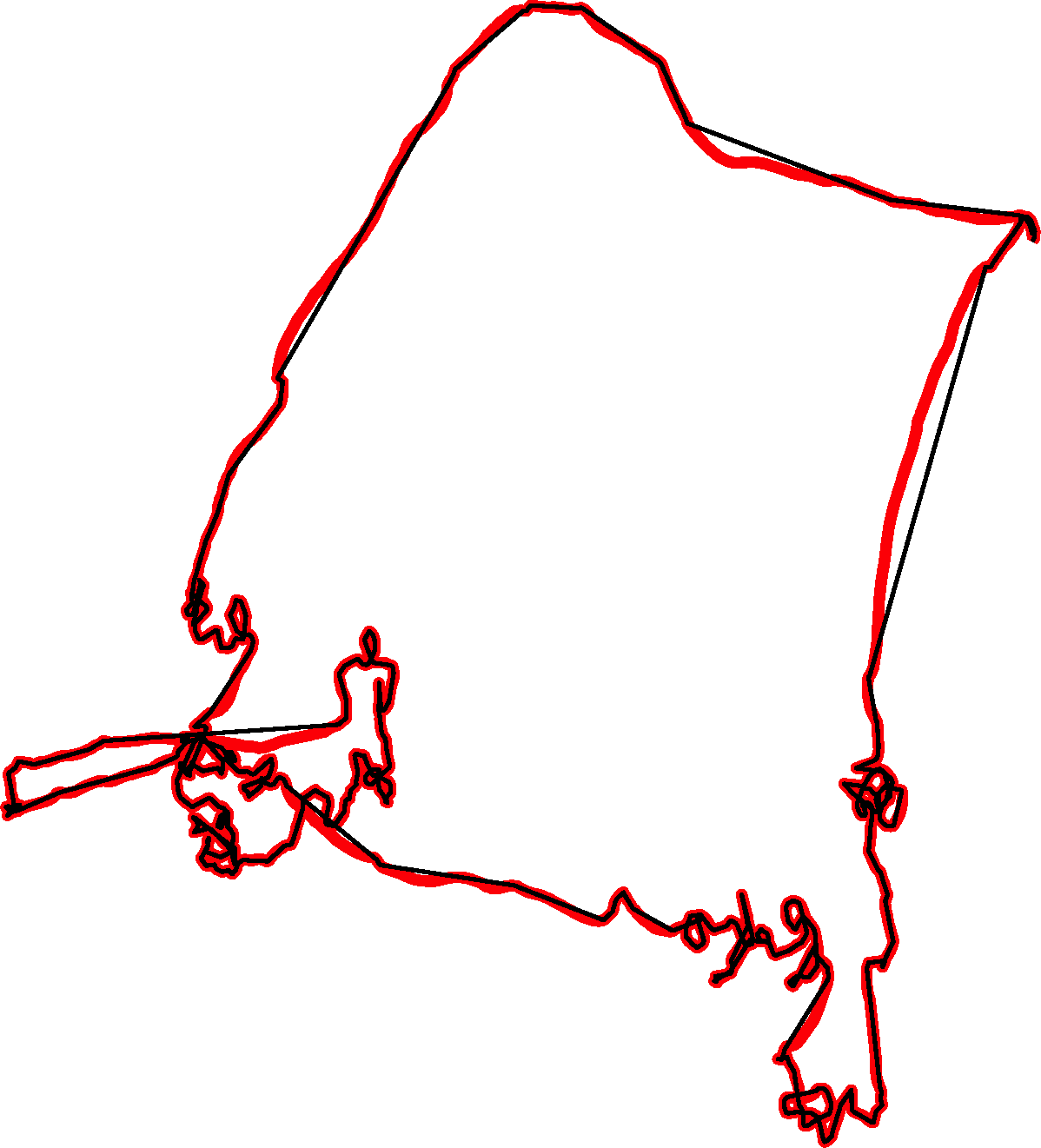

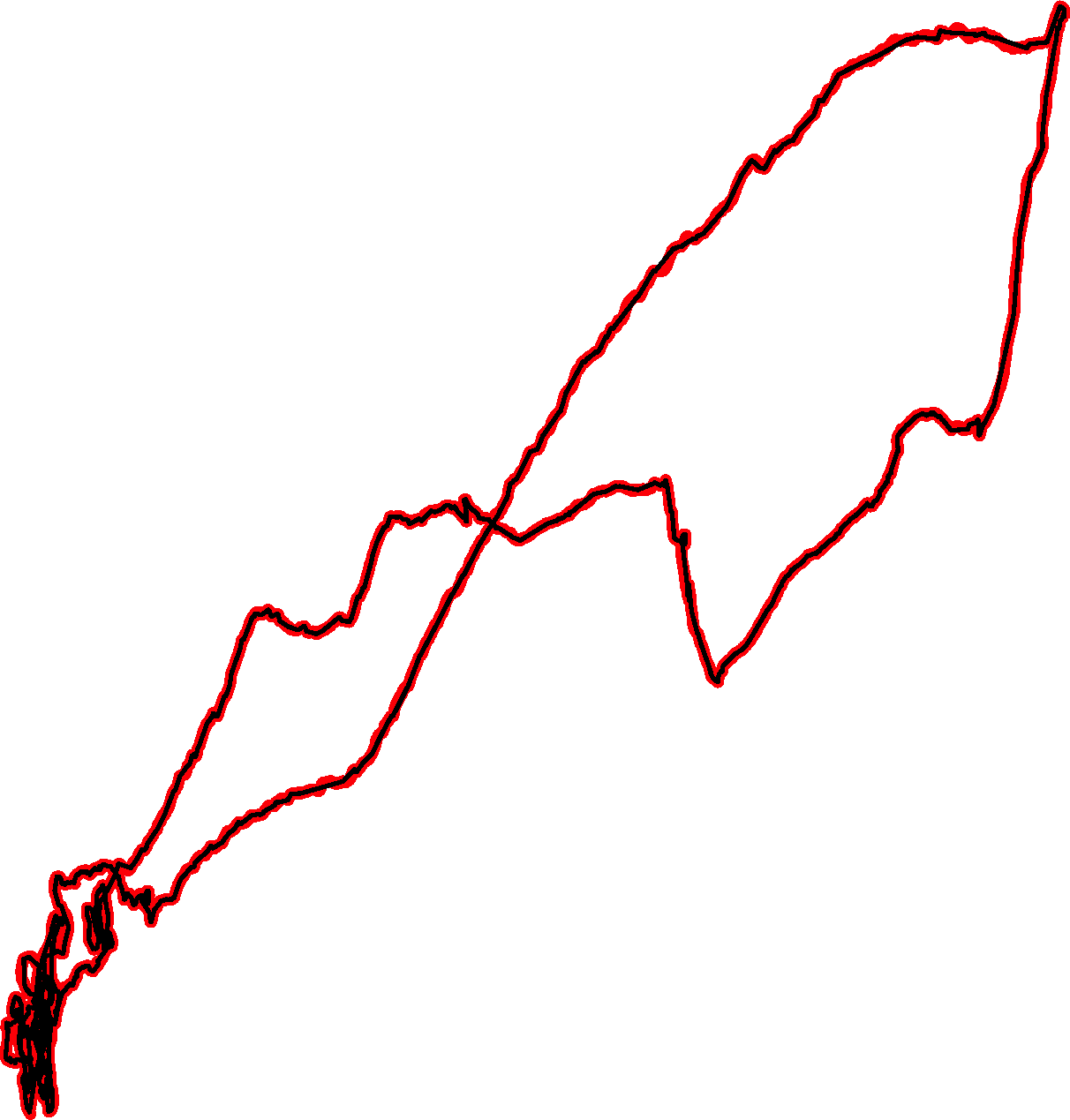


a)

d)

c)

b)

**Figure 2. 1s GPS seabird tracks, original positions in red, resampled positions in black.** Track a) had 8883 original positions resampled into 2006 moves; track b) had 1901 original positions resampled into 921 moves; track c) had 4257 original positions resampled into 2654 moves; track d) had 2307 original positions resampled into 1478 moves.
